# Modulation of dlPFC function and decision-making capacity by repetitive transcranial magnetic stimulation in methamphetamine use disorder

**DOI:** 10.1038/s41398-024-03000-z

**Published:** 2024-07-08

**Authors:** Qingming Liu, Huimin Cui, Jiali Li, Ying Shen, Lei Zhang, Hui Zheng

**Affiliations:** 1 Center for Brain, Mind and Education, Shaoxing University, Shaoxing, 312000 China; 2 Department of Psychology, Shaoxing University, Shaoxing, 312000 China; 3 School of Psychology, Nanjing Normal University, Nanjing, 210024 China; 4 Rehabilitation Medicine Center, The First Affiliated Hospital of Nanjing Medical University, Nanjing, 210029 China; 5 School of Early-Childhood Education, NanJing XiaoZhuang University, Nanjing, 211171 China; 6 Shanghai Key Laboratory of Psychotic Disorders, Brain Health Institute, National Center for Mental Disorders, Shanghai Mental Health Center, Shanghai Jiao Tong University School of Medicine, Shanghai, 200030 China

**Keywords:** Addiction, Human behaviour

## Abstract

This study explores the impact of repetitive transcranial magnetic stimulation (rTMS) on decision-making capabilities in individuals with methamphetamine use disorder (MUD), alongside potential underlying psychological mechanisms. Employing the Iowa Gambling Task (IGT) and computational modeling techniques, we assessed the decision-making processes of 50 male MUD participants (24 underwent rTMS treatment, 26 received no treatment) and 39 healthy controls (HC). We compared pre- and post-rTMS treatment alterations in the left dorsolateral prefrontal cortex (dlPFC). Results revealed inferior performance in the IGT among the MUD group, characterized by aberrant model parameters in the Value-Plus-Perseverance (VPP) model, including heightened learning rate, outcome sensitivity, and reinforcement learning weight, alongside diminished response consistency and loss aversion. RTMS treatment demonstrated efficacy in reducing craving scores, enhancing decision-making abilities, and partially restoring normalcy to certain model parameters in the MUD cohort. Nonetheless, no linear relationship between changes in model parameters and craving was observed. These findings lend support to the somatic marker hypothesis, implicating the dlPFC in the decision-making deficits observed in MUD, with rTMS potentially ameliorating these deficits by modulating the function of these brain regions. This study not only offers novel insights and methodologies for MUD rehabilitation but also underscores the necessity for further research to corroborate and refine these findings. Trial Registration www.chictr.org.cn Identifier: No. ChiCTR17013610.

## Introduction

Individual with methamphetamine use disorder (MUD) commonly exhibit prominent and enduring deficits in executive function, episodic memory and information processing [1]. These cognitive functions heavily rely on intact cortical functioning, yet patients with MUD often exhibit deficiencies across various domains including working memory, memory recall, psychomotor skills, response inhibition, task-switching abilities, and risk assessment [2]. These deficiencies may have contributed at least in part to continued drug use and potentially unhealthy decisions. UNODC’s 2020 World Drug Report had shown that an unprecedented amount of amphetamine was confiscated worldwide, dominated by methamphetamine, which represented a 16% increase from the previous year, and the geographical scale of methamphetamine manufacture and use also continues to spread [3]. Two studies posit irrational decision-making as both an outcome and a crucial risk factor for persistent methamphetamine dependence [4, 5]. The decision to opt for the short-term dopamine surge offered by methamphetamine [6], while disregarding its long-term negative implications [7], exemplifies such irrationality. Traditional theories propose that the frontal lobes primarily govern decision-making [8]. In patients with MUD, this symptom is believed to stem from irregularities in striatal dopamine pathways [9]. Current evidence indicates that repetitive transcranial magnetic stimulation (rTMS) of the dorsolateral prefrontal cortex (dlPFC) may affect processes linked to substance use disorders [10, 11].

Repetitive transcranial magnetic stimulation (rTMS), a transcranial magnetic stimulation (TMS) mode, continually stimulates the cerebral cortex through numerous repeated magnetic stimulation pulses, thereby regulating the excitability of cerebral cortex neural tissues, emerges as a ground-breaking tool for modulating prefrontal neural activity and intervening in patients with MUD, particularly in addressing cravings. Craving, a common symptom occurrence in individuals with substance use disorders, significantly heightens relapse risk [12]. Studies have demonstrated rTMS’s positive impact on cravings and relapse prevention [13]. However, previous evidences inadequately explore the psychological mechanisms through which rTMS operates. Individual decision-making behavior holds promise as a potential therapeutic indicator for substance use disorders and may also influence cravings. Recent neuroscientific studies have revealed rTMS’s capacity to modulate human behavior and cognition, effectively intervening in individual decision-making processes [14, 15]. When individuals make behavioral choices and decisions, they involve value assessment and self-control, in which the DLPFC is closely associated [16]. While the connectivity between the dlPFC and subcortical brain regions is believed to be linked to cravings, current evidence predominantly supports network formation in cortical brain regions targeted by dlPFC interventions [17], highlighting the significance of dlPFC localization. Future studies should focus on influencing subcortical brain nuclei through dlPFC stimulation. In conclusion, we posit that applying rTMS intervention to the dlPFC in our study may influence individuals’ decision-making processes. The dlPFC plays an integral role in the executive system and cognitive control of behaviors, including decision-making in substance-dependent individuals. This cognitive control impacts individuals’ spontaneous and cue-induced cravings [14, 18].

High-frequency rTMS in the dlPFC can enhance neuronal excitability in this region, therefore enhancing executive control to reduce drug cravings. Drug users exhibit significantly lower dlPFC activation than healthy individuals [19]. For example, greater activation in the OFC in cocaine abusers compared to a control group may reflect differences in the anticipation of reward while less activation in the DLPFC and MPFC may reflect differences in planning and working memory [20]. High-frequency rTMS in the left dlPFC can enhance neuronal excitability in this region [15], thereby bolstering decision-making abilities and performance on decision-making tasks. Existing research suggests that high-frequency rTMS (HF-rTMS) over the left dlPFC may decrease cravings and impulsivity in patients with MUD, aiding them in avoiding risky decisions [21, 22] and improving decision-making function [22]. Studies of resting-state functional connectivity and electroencephalogram reveal that rTMS onset of action may be through the cortex affecting deep nuclei [23, 24]. TMS interventions can stimulate cortical-striatal-thalamic-cortical circuits between the dlPFC region and the dorsal striatum, thalamus, and default mode networks, thereby influencing participants’ value judgments and decision-making abilities [25]. Nevertheless, little research investigates how patients’ decision-making processes undergo change.

Users of several central nervous system stimulants, including stimulants such as amphetamines, methcathinone, and ecstasy, are recognized for poor decision-making [26, 27] and associated altered patterns of prefrontal area activation [28]. The Iowa Gambling Task (IGT), a classical decision-making task used in over 400 studies to gauge frontal lobe damage (especially in the ventromedial prefrontal cortex (vmPFC)) across numerous clinical populations [29], is noteworthy. In the IGT, healthy participants adeptly identify and adhere to the advantageous deck after multiple selections, whereas patient’s dependent on substances consistently opt for choices offering immediate gains, despite the likelihood of greater future losses [30]. In contrast to the classic delay discounting task and balloon simulation task, the IGT task involves uncertainty, a balance of reward and punishment, and is thought to be directly related to prefrontal damage. The Somatic Marker Hypothesis (SMH) suggests that decision-making is a synergy of emotional and cognitive processing [31]. Moreover, the IGT’s solitaire-like task model enhances ecological validity and encourages task engagement, yet traditional analytical methods merely capture overall decision outcomes, lacking specificity in elucidating the underlying decision-making processes. To refine this understanding, computational modeling methods have been introduced. Several models, including the prospective value-learning model with the Delta rule (PVL-Delta) and the value-plus-perseverance (VPP) model, have been proposed to address the IGT’s specificity shortcomings [32–34]. This modeling approach has been applied to several clinical populations [35]. Specifically, the prospective value-learning model with the Delta rule (PVL-Delta) has demonstrated superior long-term prediction accuracy and parameter recovery [36, 37], while the value-plus-perseverance (VPP) model has shown excellent short-term forecasting accuracy [34, 36]. Prior studies have compared the application of these two models in assessing decision-making in patients with amphetamine use disorder [38]. Our study focuses on analyzing the decision-making process in patients with methamphetamine addiction. Further, brain imaging results reveal that several prefrontal lobe regions, including the dlPFC, medial orbitofrontal cortex (mOFC), and vmPFC, along with subcortical brain areas, including the amygdala and hippocampus, are crucial for computational processing in the IGT [39]. This region-specific aberration in risk decision-making implies that the prefrontal lobe might serve as the epicenter of the disease in MUD patients.

Consequently, this study aims to ascertain whether rTMS over the left dlPFC can reduce craving, enhance the decision-making abilities of patients with MUD, and employ model parameters to precisely measure the decision-making process. In addition, we hypothesized that: (1) real rTMS intervention will reduce cravings in MUD patients; (2) after rTMS intervention, the VPP parameters of MUD patients tended to healthy people, and the decision-making ability of patients is improved. (3) via computational model analysis of the decision-making process, rTMS intervention effects on cravings are achieved by reinstating impaired executive control in patients with MUD.

## Methods

### Participants

Our study took place from September to December 2020 in Zhejiang Province, China. With classical behavioral studies of IGT differences between addicted and healthy controls, we first need to make sure there is enough power to detect differences between the two groups [40]. Our previous studies of craving interventions have found that 10 people in the experimental group and 10 in the control group are effective [41]. We recruited 60 participants with MUD and 44 matched healthy controls (HC) group. Each participant completed all scales and the first IGT within one week. After quality control (see Section “Quality control” for details), these participants were excluded from our final dataset (10 in the MUD group and 5 in the HC group), with 50 in the MUD group and 39 in the HC group. In the MUD group, the remaining 50 patients of MUD were randomly allocated to either treatment group (MUD-t) 10 Hz rTMS (20 treatments, one per day, with five treatments followed by two days of rest, *n* = 24) or a blank group (MUD-n) who did not receive any rTMS (*n* = 26) using a computer generated number. Post-treatment, the IGT was immediately administered (only the treated MUD group (*n* = 24) received a second IGT). Informed consent was obtained from all participants. This study complied with the final version of the Declaration of Helsinki. Nanjing Normal University’s Ethics Committee approved the study protocol.

MUD group participants, (1) aged between 18 to 65 years; (2) met at least two Diagnostic and Statistical Manual of Mental Disorders, Fifth Edition (DSM-5) criteria for MUD in the preceding 12 months. Both the MUD group and controls were excluded (1) if they had a history of other mental health diagnoses; (2) scored higher than 3 on the Fagerström Tolerance Questionnaire (FTQ); or (3) scored higher than 8 on the Alcohol Use Disorders Identification Test (AUDIT); (4) Other exclusion criteria included investigator discretion based on unsuitability for participation in the study for other reasons.

### rTMS treatment procedure and craving measure

**Fig. 1:**
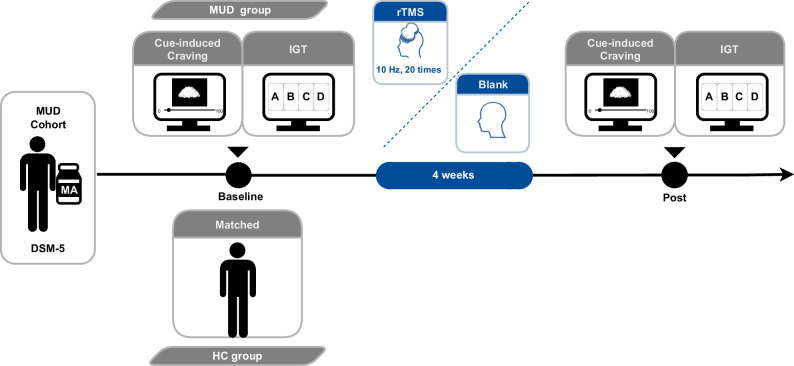
Flowchart of this study. A total of 60 patients with methamphetamine use disorder (MUD) and 44 healthy controls were recruited, as originally planned, of which 30 received the 10-HZ rTMS intervention (MUD-t) and the other 30 served as blank controls (MUD-n). However, because of quality control, 4 of them left the facility without completing the intervention due to criminal offenses, and another 6 MUD patients and 5 HC failed the quality control of the IGT task not to be included in the data analysis (more details show in the Methods section), and finally a total of 24 completed the intervention and 26 served as blank controls.

Figure 1 outlines the rTMS treatment procedure. Based on DSM-5 criteria, stimulant use disorder (MUD) diagnoses were established after consent was obtained. The IGT was then performed on a provided computer in a quiet room before and after 20-day rTMS treatment. The craving scale was then completed by the participants. Before and after rTMS treatment, craving for MUD was measured on a craving scale ranging from 1 to 100 [21]. MUD was treated with HF-rTMS. During the 20-day rTMS treatment protocol, the left dlPFC was stimulated once a day, five days a week, two days apart, with a frequency of 10 Hz, a pulse intensity of 100% of resting motor threshold, and a duration of 5 s, interval of 10 s, 40 repetitions, and 2000 pulses. The stimulation intensity was adjusted according to participant tolerance. The resting motor threshold (RMT) was individually determined based on a 50% probability (5 out of 10 trials) of contraction in the index finger muscle (FDI). Consistent with our previous research, the left DLPFC was located using a positioning cap, with a circular coil from CCY-IA TMS device (Wuhan Yiruide Biotechnology Co., Ltd. Wuhan, China) [42].

### Iowa gambling task

The original IGT version [43] presents deck A and B as “bad” decks owing to their negative long-term expected value, while deck C and D are “good” decks with a positive long-term expected value. We maintained a predetermined card order within each deck, ensuring a uniform sequence of outcomes for each participant (Fig. 1). The task was conducted in a soundproof room, keeping constant light and a comfortable temperature. The distance between the computer screen and the participant’s eyes was set at 70 cm. We used E-Prime 2.0 software (Psychology Software Tools) to program the experiment. The computer screen’s refresh rate was 60 Hz, with a resolution of 1024 × 768 pixels and a viewing distance of 60 cm. During the entire experiment, participants were instructed to gaze at the screen’s center and keep their bodies relaxed.

Specifically, each participant was required to complete 100 (divided into five blocks) selections, and for each selection, the participant had to choose one card out of four stacks (A–D). The goal is to win as much money as possible and lose as little money as possible. The players had 2,000 RMB of game principals. The player will choose any of the stacks and simply flip the cards, knowing with each flip whether they are winning a certain amount or losing a certain amount, and seeing how much of their current principal they have. Practically, each stack of cards represents the following:

- Card A: Win 100 RMB and may lose a certain amount (150, 200, 250, 300, 350, 10% probability)
- Card B: Win 100 RMB and 10% probability of losing 1250 RMB
- Card C: Win 50 RMB and may lose (25 12.5%, 50 25%, 75 12.5%)
- Card D: Win 50 RMB and 10% probability of losing 250 RMB

However, the probability law corresponding to A/B/C/D is not known to the player in advance and can only be perceived by trying.

### Quality control

Our data underwent filtering based on two primary criteria. Firstly, we verified participants’ understanding of the task and their motivation to garner more rewards, each participant completed an exercise designed to familiarize them with the selection process. No exclusions occurred at this step. Four MUD patients were excluded from data analysis because they were arrested after post-testing due to involvement in other criminal offenses, and they left the rehabilitation center. Secondly, the task demanded exploratory learning across all options; thus, subjects who selected an option less than 5% of the time (five times or less) were considered to lack sufficient data for our study (6 in MUD and 5 in HC group) [44]. Consequently, these participants were excluded from our final dataset (10 participants from the MUD group and five from the HC group). After this quality control process, we had 50 male participants (average age *M* = 36.20, *SD* = 9.96) in the MUD group and 39 male participants (average age *M* = 35.34, *SD* = 6.81) in the HC group, with *t* (87) = 0.472, *p* = 0.783, *d* = 0.101. Table 1 provides further details on the subgroups.

**Table 1:** The demographic variables in this study.

	HC M (SD)	MUD-t M (SD)	MUD-n M (SD)	*F/t*	*p*	Effect size
Gender (Female/Male)	0/39	0/24	0/26	/	/	/
Age	36.2 (9.96)	34 (7.62)	36.5 (5.85)	0.673	0.513	0.015
Addiction year	/	8.75 (3.42)	9.54 (3.19)	0.844	0.403	0.2389
Usage (dosage*time^−1^)	/	0.94 (0.50)	0.91 (0.38)	−0.273	0.786	−0.0774
Usage (dosage*month^−1^)	/	15.83 (13.54)	16.19 (8.88)	0.112	0.912	0.0316
Craving	/	64.58 (17.69)	67.31 (17.79)	0.542	0.59	0.1536

*MUD-t* methamphetamine use disorder with rTMS treatment, *MUD-n* methamphetamine use disorder with non-rTMS treatment, effect size *η*^2^*p* or cohen’s *d*.

### IGT model framework

Drawing from past literature, we integrated four models: Outcome-Representation Learning Model (ORL), Prospect Valence Learning (PVL) decay-RI, Prospect Valence Learning (PVL) Delta, and Value-Plus-Perseverance (VPP) [38]. The VPP model emerged as the winner, prompting us to focus on its formulas and parameters. This model consists of eight free parameters (*A*, *α*, cons, *λ*, *ε*_*p*_, *ε*_*n*_, *K*, *ω*), grouped into three parts: utility functions (*α*, *λ*), value update rules (*A*, *ω*), and action selection rules (cons, *ε*_*p*_, *ε*_*n*_, *K*).

The EV utility function assumes differential weighting of gains and losses. The parameter *w* (0 ≤ *w* ≤ 1) represents the extent to which participants weigh gains against losses. A value over 0.50 indicates a gain weight higher than the loss weight. The parameter *t* denotes the current trial.

1 $$u\left(t\right)=w* {\rm{win}}\left(t\right)-\left(1-w\right)* {\rm{loss}}\left(t\right)$$

The prospect valence utility function posits that each outcome’s evaluation follows a utility function derived from the prospect theory [32], generally less sensitive to amplitude increases and more sensitive to losses. Here, *α* is the shape parameter (0 < *α* < 2), and *λ* is a loss aversion parameter (0 < *λ* < 5) denoting sensitivity to gain and loss. A *λ* value over 1 signifies greater sensitivity to loss than gain.

2 $$u\left(t\right)=\left\{\begin{array}{c}x{\left(t\right)}^{\alpha },{{\rm{if}}\,x}(t)\ge 0\\ -\lambda {\left|x\left(t\right)\right|}^{\alpha },{{\rm{if}}\,x}\left(t\right) \,<\, 0\end{array}\right.$$

The Value-updating rule serves to update the expected values or expectancies *E*_*i*_(*t*) for the chosen option *i* in trial *t*. Based on the delta rule, the expected value is the newly weighted average of rewards received for each option. Here, *φ* (0 ≤ *φ* ≤ 1) illustrates the recent results’ weighting when updating expectations. Higher *φ* values suggest a stronger weighting of recent outcomes.

3 $${E}_{i}\left(t\right)={E}_{i}\left(t-1\right)+\varphi * \left[u\left(t\right)-{E}_{i}\left(t-1\right)\right]$$

The Decay rule assumes that all decks decay or discount over time and then adds the selected decks’ expected values to the current outcome utility. The parameter *A* (0 ≤ *A* ≤ 1) decides the discount for past expected values, and ${\delta }_{i}\left(t\right)$ is a dummy variable, set to 1 if deck j is selected and 0 if otherwise.

4 $${E}_{i}\left(t\right)=A* {E}_{i}\left(t-1\right)+{\delta }_{i}\left(t\right)* u\left(t\right)$$

The action selection rule utilizes a softmax rule [45], determining the predicted probability of selecting deck *j* in trial *t*, Pr[G_*j*_(t)].

5 $$\Pr \left({G}_{j}\left(t\right)\right)=\frac{{{\rm{e}}}^{\theta \left(t\right)}* {E}_{j}\left(t\right)}{{\sum }_{j={j}^{{\rm{e}}}}^{4}\left|\theta \left(t\right)* {E}_{j}\left(t\right)\right|}$$

The parameter cons (0 ≤ *c* ≤ 5) signifies response consistency or exploitation parameters [46]. A higher cons value shows a stronger preference for choosing an option with a higher expected value, while a lower value indicates a higher propensity for exploring options with a lower expected value.

6 $$\theta (t)={3}^{\rm{cons}}-1$$

The VPP model introduces three additional free parameters (*K*, *ε*_*p*_, *ε*_*n*_) in the Action-selection rule. *K* (0 < *K* < 1) is a decay parameter, similar to *A* in the PVL-DecayRI model, deciding the decay rate of the persistence strength of all decks (including unselected decks) on each trial *t*. *ε*_*p*_ and *ε*_*n*_ represent the effects of gains and losses on adherence behavior, respectively. Positive values suggest feedback strengthens the tendency to stick with the same deck in the subsequent trial, whereas negative values denote feedback bolstering the inclination to switch from the selected deck. The VPP model assumes that participants track deck expectations *E*_*j*_(*t*) and perseverance intensity *P*_*j*_(t). Expectations were calculated using the learning rule of the PVL-Delta model (Eq. 4). For the perseverance advantage of the unselected set on the current trial *t*, *P*_*j*_(*t*) = k * *P*_*j*_(*t* *−* 1).

7 $${P}_{j}(t)=\left\{\begin{array}{c}k* {P}_{j}(t-1)+{\varepsilon }_{p},{{\rm{if}}\,x}(t)\ge 0\\ k* {P}_{j}(t-1)+{\varepsilon }_{n},{{\rm{if}}\,x}\left(t\right) \,<\, 0\end{array}\right.$$

The parameter ω is added to the Value-update rule. The total value of *V*_*j*_ (*t*) is the weighted sum of *E*_*j*_ (*t*) and *P*_*j*_ (*t*), with ω denoting the weight of the RL (0 < *ω* < 1). Lower *ω* values indicate that participants depend less on RL and more on the perseverance heuristic, and vice versa. High values of ω indicate that subjects rely more on RL and less on the perseverance heuristic. In the VPP model, the selection probabilities are once again used with the softmax rule but using *V*_*j*_ (*t*).

8 $${V}_{j}(t)=\omega \cdot {E}_{j}(t)+(1-\omega )\cdot {P}_{j}(t)$$

### Statistical analysis

For model fit assessment, we employed the hierarchical Bayesian modelling package (hBayesDM version 1.1.1) [47] in R Core (4.1.3). We initially fitted the ORL, PVL Decay-RI, and PVL Delta models to the data, selecting the best model using the Leave-One-Out Information Criterion (LOOIC) or the widely applicable information criterion (WAIC) for comparison [48]. Additional tests (e.g., posterior prediction plots) and visualizations (e.g., Markov Monte Carlo chain [MCMC] plots) will validate our confidence in the model results. We will express concerns if the MCMCs do not converge, as discernible from trace plots for all parameters of each model. We provide further details on these processes in the Table S1.

For parameters and clinical factors, we employed several statistical analysis approaches based on the NHST. We will interpret p-values below the set alpha level of <0.05 as indicative of statistical significance. Independent t-tests and chi-square tests were employed for comparisons between the addiction and HC groups. In the present study, covariates that needed to be included were no longer considered as there were no statistical differences between the healthy and patient groups. In the treatment ANOVA statistics, only indicators related to addiction could be used as covariates, but they were not included in the analysis because they did not show a statistical difference between the groups (as shown in Table 1), as well as there was no need for a post-hoc side. Paired t-tests and repeated-measures ANOVA were used to evaluate the rTMS effects. Pearson’s correlation coefficient was utilized to examine the correlation between parameters and clinical factors. We also provided Cohen’s *d* and partial *η*^2^ for statistical effect. All analyses were executed using the R-based statistical software Jamovi (version 2.2.5). Additionally, we used dabestr (version 0.3.0) in R (version 4.1.3) to present the effect sizes of the independent sample *t*-test and paired *t*-test [49].

## Results

### Cravings in methamphetamine addiction reduced by rTMS treatment

**Fig. 2:**
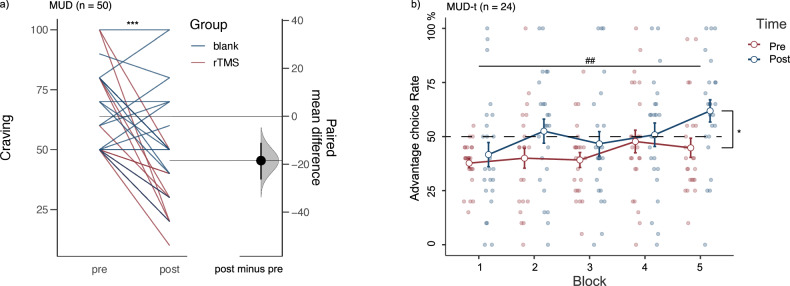
Treatment effects in the MUD group. The paired sample *t*-test and repeated-variance test showed improvement in the MUD group. **a** A line graph shows a significant reduction in craving in the MUD treatment (MUD-t) group (*n* = 24), but no such change in the MUD blank (MUD-n) group (*n* = 26). Each line represents a subject’s pre- and post-treatment measurements. Post data for four MUD patients were missing from the data analysis due to arrests prior to post-testing and some patients have the same pre- and post-thirst scores, so the lines will overlap. **b** Line and scatter plots illustrate a more advantageous selection in Iowa Gambling Task for the MUD-t group after rTMS treatment. Dark red lines represent pre-rTMS patients, while dark blue indicates post-rTMS patients. The proportion of beneficial deck selection increases as the experimental blocking progresses (***p < 0.001, **p < 0.01, *p < 0.05, ^##^ means that the main effect of block is significant, specifically *p* < 0.01).

To assess the immediate effects of rTMS treatment, we implemented a mixed ANOVA for cue-induced craving. The mixed ANOVA, with Time (pre-, post-) as within-subjects’ factor and Group (MUD-t, MUD-n) as between-subjects’ factor, revealed significant main effects of Time (*F* (1, 54) = 45.31, *p* < 0.001, *η*_*p*_^2^ = 0.46) and Group (*F* (1, 54) = 6.2, *p* = 0.016, *η*_*p*_^2^ = 0.10), and a significant interaction between Time and Group (*F* (1, 54) = 21.04, *p* < 0.001, *η*_*p*_^2^ = 0.28). The simple effect demonstrated a significant decrease in the craving score post-intervention (*M* = 34.6, *SD* = 13.8) compared with pre-intervention (*M* = 66.0, *SD* = 17.6, *t* (24) = 8.31, *p* < 0.001, *d* = 1.70). The MUD-n showed no significant differences (Fig. 2a and Tables S2 and S3).

To assess the altered of IGT performance of rTMS treatment, we implemented a repeated-measures ANOVA for the advantageous choice rate in the IGT for the MUD-t group. The 2 × 2 repeated-measures ANOVA, with Time (pre-, post-) and Block (1–5) as within-subjects’ factors, revealed a main effect of Time (*F* (1, 23) = 6.22, *p* = 0.020, *η*_*p*_^2^ = 0.213) and Block (*F* (4, 92) = 4.12, *p* = 0.004, *η*_*p*_^*2*^ = 0.152). There was no interaction between Time and Block (*F* (4, 92) = 1.31, *p* = 0.271, *η*_*p*_^*2*^ = 0.054; Fig. 2b). These results suggest that rTMS might alter the judgement of advantageous choices.

### Abnormal VPP parameters in methamphetamine addiction

**Fig. 3:**
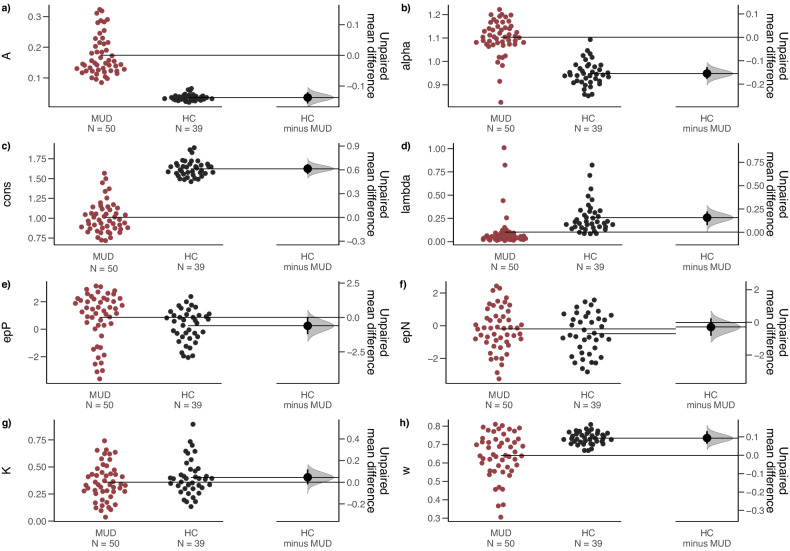
Abnormal VPP parameters in the MUD group. Gardner–Altman plots were used to show the difference between two groups (MUD (*n* = 50) vs. HC (*n* = 39)). Red solid dots represent the parameters for each individual in the MUD group (jitter), while black solid dots represent the parameter scores for the healthy control group. The funnel-shaped gray range on the right indicates the difference between the two groups, with the large black dot showing the mean of the difference, and the short black vertical line representing the 95% confidence interval of the difference. A confidence interval that does not cross zero signifies a statistically significant difference between the two groups. This suggests that the MUD group had significantly higher values for parameters *A* and alpha, and significantly lower values for parameters cons, lambda, and ${\rm{\omega }}$.

To assess the different between the MUD group and HC group, independent samples t-tests were applied to test the IGT model parameters for decision irregularities in the MUD group (Fig. 3 and Figs. S1, 2, 4, 5, 8, and 9). The test revealed several parameters significantly different between the MUD and HC groups. Compared with the HC group (*M* = 0.04, *SD* = 0.01), the MUD group had a significantly higher learning rate (A) (*M* = 0.17, *SD* = 0.07) (*t* (87) = −13.04, *p* < 0.001, *d* = −2.786, Figure S7), and the outcome sensitivity (alpha) in the MUD group (*M* = 1.1, *SD* = 0.07) was also significantly higher than that in the HC group (*M* = 0.95, *SD* = 0.05) (*t* (87) = −11.17, *p* < 0.001, *d* = −2.385). Conversely, the MUD group demonstrated significantly lower response consistency (cons) (*M* = 1.01, *SD* = 0.2) than the HC group (*M* = 1.62, *SD* = 0.1) (*t* (87) = 17.86, *p* < 0.001, *d* = 3.815), and loss aversion (lambda) (*M* = 0.1, *SD* = 0.18) was significantly lower than that in the HC group (*M* = 0.26, *SD* = 0.16) (*t* (87) = 4.19, *p* < 0.001, *d* = 0.896). The MUD group’s reinforcement learning weight (w) (*M* = 0.64, *SD* = 0.12) was also significantly lower than the HC group’s (*M* = 0.74, *SD* = 0.03) (*t* (87) = 4.74, *p* < 0.001, *d* = 1.013; see Table 2 for details). No between-group differences were found in the pre-tests between the treated and untreated groups in the MUD group (*p*_*s*_ > 0.05, See Tables S2, S3). These results highlight an impaired IGT performance in the MUD group compared with healthy controls.

**Table 2:** The parameters for VPP model between MUD group and HC group.

	HC M (SD) *n* = 39	Meth M (SD) *n* = 50	*t*	d*f*	*p*	Choen’s *d*
*A*	0.04 (0.01)	0.17 (0.07)	−13.04^a^	87	<0.001	−2.786
alpha	0.95 (0.05)	1.1 (0.07)	−11.17	87	<0.001	−2.385
cons	1.62 (0.1)	1.01 (0.2)	17.86^a^	87	<0.001	3.815
lambda	0.26 (0.16)	0.1 (0.18)	4.19	87	<0.001	0.896
epP	0.25 (1.18)	0.86 (1.81)	−1.83^a^	87	0.071	−0.391
epN	−0.5 (1.23)	−0.21 (1.31)	−1.06	87	0.292	−0.226
*K*	0.4 (0.17)	0.36 (0.17)	1.29	87	0.201	0.275
*ω*	0.74 (0.03)	0.64 (0.12)	4.74^a^	87	<0.001	1.013
	**Pre** **M (SD)** ***n*** = **24**	**Post** **M (SD)** ***n*** = **24**				
*A*	0.19 (0.08)	0.05 (0.002)	9.33	23	<0.001	1.904
alpha	1.09 (0.09)	0.71 (0.05)	16.59	23	<0.001	3.386
cons	1.01 (0.18)	1.14 (0.09)	−2.93	23	0.008	−0.598
lambda	0.13 (0.25)	0.51 (0.15)	−8.1	23	<0.001	−1.653
epP	0.62 (1.89)	1.03 (0.76)	−1.17	23	0.254	−0.239
epN	−0.28 (1.23)	0.21 (1.05)	−2.57	23	0.017	−0.525
*K*	0.34 (0.16)	0.47 (0.15)	−3.23	23	0.004	−0.659
*ω*	0.65 (0.11)	0.54 (0.01)	4.83	23	<0.001	0.985

*HC* health control group, *MUD* methamphetamine use disorder group, *Pre* methamphetamine use disorder group before the rTMS treatment, *Post* methamphetamine use disorder group after the rTMS treatment, *A* learning rate, *alpha* outcome sensitivity, *cons* response consistency, *lambda* loss aversion, *epP* gain impact, *epN* loss impact, *K* decay rate, *ω* reinforcement learning weight.

^a^Levene’s test is significant (*p* < 0.05); suggesting a violation of the assumption of equal variances.

### Improvement in abnormal VPP parameters in the MUD group via rTMS treatment

**Fig. 4:**
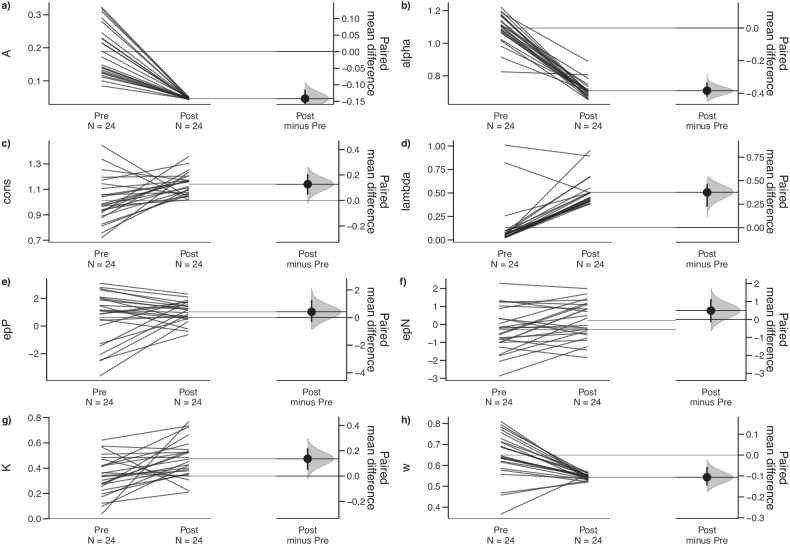
rTMS treatment improves abnormal VPP parameters in the MUD group. Pre- and post-intervention difference in the MUD treatment (MUD-t) group (*n* = 24) were showing by Gardner–Altman plots. Black solid lines represent the parameters for pre- and post-rTMS measurements in the MUD-t group. The funnel-shaped gray range on the right signifies the difference between the two time points, with the large black dot indicating the mean of the difference, and a short black vertical line representing the 95% confidence interval of the difference. A confidence interval that does not cross zero indicates a statistically significant difference between the two groups. This suggests that rTMS significantly decreased parameters *A*, alpha, and ω, and significantly increased parameters cons, lambda, and *K*.

A paired-sample *t*-test for the IGT model parameters was employed to determine the direct effect of rTMS treatment (Figs. 4, S3, 6, and 10) for MUD-t group (n = 24). In general, MUD group treated with rTMS showed decreased learning rates (*t* (23) = 9.33, *p* < 0.001, *d* = 1.904), outcome sensitivity (*t* (23) = 16.59, *p* < 0.001, *d* = 3.386), and reinforcement learning weight (*t* (23) = 4.83, *p* < 0.001, *d* = 0.985), while experiencing significant increases in response consistency (*t* (23) = −2.93, *p* = 0.008, *d* = −0.598), loss aversion (*t* (23) = −8.1, *p* < 0.001, *d* = −1.653), loss impact (*t* (23) = −2.57, *p* = 0.017, *d* = −0.525), and decay rate (*t* (23) = −3.23, *p* = 0.004, *d* = −0.659) (see Table 2 for details).

### Correlation of abnormal parameters and clinical indicators in the MUD group

To explore the relationship between abnormal decision-making and the clinical symptoms of addiction, we conducted a Pearson correlation analysis. Within the MUD group (*n* = 50), the weight of reinforcement showed a positive correlation with learning with the dose per use (g) (*r* (48) = 0.325, *p* = 0.021), but not addiction year (*r* (48) = −0.112, *p* = 0.439), dose per month (g) (*r* (48) = 0.167, *p* = 0.246), craving (*r* (48) = −0.093, *p* = 0.523). The delta craving [pre–post] in MUD-t group (*n* = 24) was positive correlation with weight of reinforcement (*r* (22) = 0.115, *p* = 0.592), years of addiction (*r* (22) = 0.452, *p* = 0.026), pre-test craving (*r* (22) = 0.695, *p* < 0.001) but not dose per time (g) (*r* (22) = 0.257, *p* = 0.226) and dose per month (g) (*r* (22) = 0.361, *p* = 0.083). This implies a potential non-linear relationship between changes in decision-making abilities and changes in addictive craving.

## Discussion

This study employed a computational modeling approach to elucidate the decision-making psychological mechanisms underlying rTMS intervention on patients with MUD. Consistent with prior research [50–53], rTMS treatment targeting the left dlPFC resulted in decreased self-reported craving among MUD patients. A recent study demonstrated that changes in EEG microstates in patients with MUD were linked to a reduction in craving levels after receiving rTMS [54]. Moreover, model parameters revealed abnormalities in the decision-making processes of MUD group compared to HC group, which rTMS treatment mitigated, thereby improving decision-making capabilities. Specifically, sensitivity to outcomes and reinforcement learning rates were altered in MUD-t group post-treatment. While these findings were aligned with our expectations, contrary to our hypothesis, no significant correlation between changes in decision-making parameters and cravings was observed. This suggests potential independence between craving reduction and decision-making improvement post-rTMS intervention, warranting innovative ideas for future intervention programs.

Notably, some model parameters in the MUD group deviated from HC group, with certain parameters reverting to healthy levels following rTMS intervention. Within the utility function, α shapes the function, while a larger λ indicates stronger loss aversion. MUD patients exhibited reduced loss aversion, which rTMS intervention increased, aligning it more closely with healthy individuals. Regarding value update, *A* reflects the influence of prior round’s expected value on the current round. Unlike HC group, MUD group demonstrated a strong eligibility trace effect pre-intervention, which normalized post-treatment. In action selection, lower cons values indicate greater exploration tendency. The MUD group’s cons value increased after treatment, indicating a stronger tendency towards rational decision-making based on existing information. No significant difference between the HC and MUD groups was found for *ε*_*p*_ and *ε*_*n*_, thereby confirming the model’s reliability as these parameters did not change significantly post-intervention. Although the most appropriate model for the present study differed from that obtained by previous researchers in the amphetamine study, the results regarding reward/outcome sensitivity were consistent, both in that centrally excitatory substance use disorder showed higher reward sensitivity (alpha) relative to HC group [36]. In particular, we found that this elevated outcome sensitivity for MUD group was reversed after the rTMS intervention, and that alpha may be a potential computational indicator regarding addiction recovery.

However, some parameters exhibited different alterations compared to HC group post-rTMS intervention. In the value-update section, lower *ω* reflects greater reliance on previous response tendencies when choosing. MUD group’s *ω* was lower but increased post-treatment, suggesting enhanced dependency on previous selection tendencies. We attribute this to changes in *K* (choice decay), as evidenced by increased *K* post-intervention, influencing final value updates.

This study discovered that the MUD group’s VPP parameters were abnormal, signifying compromised decision-making capabilities, a conclusion congruent with earlier studies [55–57]. The SMH proposes that daily decision-making reflects the balancing of options [31]. These options, associated with positive or negative somatic states, act as expected emotional signals, predicting the rewards or punishments of a choice. Individuals with vmPFC brain injuries may fail to make decisions as they are unable to utilize emotional signals generated by their bodies to evaluate the outcomes of options.

The study’s results demonstrated that abnormal VPP parameters in the MUD group improved after rTMS treatment, enhancing patient performance in the decision-making process. Earlier research confirmed that rTMS to the left dlPFC improves decision-making abilities in patients [58], a finding corroborated by our current study. Furthermore, reinforcement learning abilities improved post-treatment, indicating improved information extraction from past experiences and superior decision-making. This supports the role of vmPFC function in decision-making among MUD group, as suggested by the SMH, where normal decision-making relies on both emotional and cognitive facets. Treatment targeting the dlPFC not only enhances decision-making abilities but also improves vmPFC function, indicating potential mutual influence between these brain regions.

Despite these insights, we found no evidence of a linear relationship between model parameter changes and alterations in addiction symptoms like craving. This could be due to experimental inaccuracies in model measurements, such as insufficiently objective craving measurements. Furthermore, parameter changes and addiction symptom shifts might not be linear, with a possible lag effect [59] suggesting that craving changes in patients do not occur simultaneously with model parameter changes.

The study has some limitations. The self-report method used to gauge cravings is subject to subjective effects, necessitating further improvement of validation. In actual measurements, most patients will tend to choose whole number craving scores, e.g., multiples of 10 such as 50, 60, etc. This may reduce the accuracy of the measurements, and it is possible that in future studies the labeling of the VAS will only be provided with a start of 0 and an end of 100. The IGT used to measure patient decision-making simulates and predicts real-world decision-making behavior, with its inherent risks and ambiguities, and a blend of gains and losses. IGT tasks were also not post-tested in the MUD-n group. The study also lacked an extended follow-up (e.g., 1-month, 3-month, 6-month) for the MUD group. Future research should assess the long-term effects of rTMS treatment on reinforcement learning and decision-making abilities to effectively gauge clinical outcomes. The therapeutic impact of rTMS in genesis patients lacks neuroimaging evidence, requiring further validation via functional magnetic resonance imaging. In addition, subsequent research endeavors ought to contemplate integrating fMRI to enhance the precision of rTMS interventions. The small sample size of this study, due to the pandemic, should be expanded in future studies to enhance the study’s significance. A single site study is indeed unrepresentative.

In conclusion, patients with MUD exhibit deficiencies in decision-making abilities. rTMS treatment targeting the dlPFC reduced craving, alleviated addiction symptoms, and improved decision-making abilities, particularly reinforcement-learning capabilities. However, the linear relationship between decision-making changes and cravings warrants further exploration. Future research should focus on increasing participant numbers, refining decision-making task models, and investigating the impact of decision-making impairments on rehabilitation.

### Supplementary information

Supplement Methods and Results

## Data Availability

The data and code were used to generate results of this study are available from the corresponding author upon reasonable request.
