# Supplementary material for: Modulation of dlPFC function and decision-making capacity by repetitive transcranial magnetic stimulation in methamphetamine use disorder
Source: Transl Psychiatry. 2024 Jul 8;14:280. doi: 10.1038/s41398-024-03000-z (PMC11231311; doi:10.1038/s41398-024-03000-z)
Supplement: Supplementary file 1 — Supplement Methods and Results [file 41398_2024_3000_MOESM1_ESM.docx]

**Supplement Methods and Results**

For the corresponding data, first we determined by comparing the Leave-One-Out Information Criterion (LOOIC) or Widely Applicable Information Criterion (WAIC) [(Vehtari et al., 2017)](https://sciwheel.com/work/citation?ids=4604910&pre=&suf=&sa=0&dbf=0) for all models that in all groups the Value-Plus-Perseverance Model is a better fit for all groups (Table S1). Within the methamphetamine group, there were no between-group differences in pretests between the treated and untreated groups (*ps*>.05, STable2).

Subsequently, we checked that the MCMC samples of each group were well mixed and converged to a smooth distribution for the diagnosis or visualization of the super (group) parameters, and the parameters were seen to fit more consistently by sampling a total of 24,000 times for 8 chains (Figure S1-3). We can check the posterior distribution by fitting the parameters to the posterior (SFig. 4-6). To compare the two groups in a Bayesian manner [(Ahn et al., 2014)](https://sciwheel.com/work/citation?ids=3930979&pre=&suf=&sa=0&dbf=0), group differences were assessed by examining the posterior distributions of the differences in the group means, and this comparison was demonstrated by the 95% Highest Density Interval (HDI) (confidence intervals without 0 represent meaningful differences, SFig. 7, 8). For parameters with differences, we also show the distribution of parameters for individuals (SFig. 9, 10).

Table S1 The model fitness for each model in each group, related to Table 2

| Group | Model | LOOIC | WAIC | LOOIC Weights | WAIC weights |
| --- | --- | --- | --- | --- | --- |
| MUD-pre | Orl | 12,990.98 | 12,890.08 | 0 | 0 |
| MUD-pre | Pvl decay | 13,660.08 | 13,593.04 | 0 | 0 |
| MUD-pre | Pvl delta | 14,431.20 | 14,346.32 | 0 | 0 |
| MUD-pre | VPP | 12,866.90 | 12,767.29 | 1 | 1 |
| MUD-post | Orl | 5,774.44 | 5,764.72 | 0 | 0 |
| MUD-post | Pvl decay | 5,888.33 | 5,852.28 | 0 | 0 |
| MUD-post | Pvl delta | 14,914.62 | 14,849.85 | 0 | 0 |
| MUD-post | VPP | 5,704.56 | 5,683.50 | 1 | 1 |
| HC | Orl | 9,921.47 | 9,873.98 | 0 | 0 |
| HC | Pvl decay | 10,235.16 | 10,183.56 | 0 | 0 |
| HC | Pvl delta | 10,482.38 | 10,429.92 | 0 | 0 |
| HC | VPP | 9,812.55 | 9,730.54 | 1 | 1 |

The most suggested model in this test is Value-Plus-Perseverance (VPP) model.

Table S2 The parameters different between MUD-t and MUD-n, related with Figure 3, and Table 2

| **Parameters** | ***t*** | **df** | ***p*** | **Choen’s *d*** |
| --- | --- | --- | --- | --- |
| *ω* | -0.4077 | 48.0 | 0.685 | -0.1154 |
| *K* | 0.8382 | 48.0 | 0.406 | 0.2373 |
| epN | 0.3926 | 48.0 | 0.696 | 0.1111 |
| epP | 0.9011 | 48.0 | 0.372 | 0.2551 |
| lambda | -1.0913 | 48.0 | 0.281 | -0.3089 |
| cons | -0.0816 | 48.0 | 0.935 | -0.0231 |
| alpha | 0.8954 | 48.0 | 0.375 | 0.2535 |
| *A* | -1.5526 | 48.0 | 0.127 | -0.4395 |

MUD-t, methamphetamine use disorder with rTMS treatment; MUD-n, methamphetamine use disorder with non-rTMS treatment; *A*, learning rate; alpha, outcome sensitivity; cons, response consistency; lambda, loss aversion; epP, gain impact; epN, loss impact; *K*, decay rate; ω, reinforcement learning weight.

Table S3 The parameters describe in MUD-t and MUD-n, related with Figure 3, and Table 2

|  |  | **Group** |  | ***n*** |  | ***Mean*** |  | ***Median*** |  | ***SD*** |  | ***SE*** |  |
| --- | --- | --- | --- | --- | --- | --- | --- | --- | --- | --- | --- | --- | --- |
| *ω* |  | MA-no |  | 26 |  | 0.6353 |  | 0.6494 |  | 0.1285 |  | 0.02521 |  |
|  | | MA-pre |  | 24 |  | 0.649 |  | 0.6435 |  | 0.1105 |  | 0.0226 |  |
| *K* |  | MA-no |  | 26 |  | 0.3775 |  | 0.3359 |  | 0.1772 |  | 0.03475 |  |
|  | | MA-pre |  | 24 |  | 0.338 |  | 0.3366 |  | 0.1552 |  | 0.0317 |  |
| epN |  | MA-no |  | 26 |  | -0.1355 |  | -0.1143 |  | 1.4057 |  | 0.27568 |  |
|  | | MA-pre |  | 24 |  | -0.283 |  | -0.3658 |  | 1.2318 |  | 0.2514 |  |
| epP |  | MA-no |  | 26 |  | 1.0790 |  | 1.5932 |  | 1.7328 |  | 0.33983 |  |
|  | | MA-pre |  | 24 |  | 0.617 |  | 1.1185 |  | 1.8908 |  | 0.3860 |  |
| lambda |  | MA-no |  | 26 |  | 0.0748 |  | 0.0538 |  | 0.0834 |  | 0.01636 |  |
|  | | MA-pre |  | 24 |  | 0.131 |  | 0.0543 |  | 0.2479 |  | 0.0506 |  |
| cons |  | MA-no |  | 26 |  | 1.0081 |  | 0.9752 |  | 0.2132 |  | 0.04182 |  |
|  | | MA-pre |  | 24 |  | 1.013 |  | 0.9729 |  | 0.1791 |  | 0.0366 |  |
| alpha |  | MA-no |  | 26 |  | 1.1119 |  | 1.1046 |  | 0.0499 |  | 0.00978 |  |
|  | | MA-pre |  | 24 |  | 1.093 |  | 1.1060 |  | 0.0922 |  | 0.0188 |  |
| *A* |  | MA-no |  | 26 |  | 0.1599 |  | 0.1465 |  | 0.0522 |  | 0.01023 |  |
|  | | MA-pre |  | 24 |  | 0.188 |  | 0.1605 |  | 0.0752 |  | 0.0153 |  |

MUD-t, methamphetamine use disorder with rTMS treatment; MUD-n, methamphetamine use disorder with non-rTMS treatment


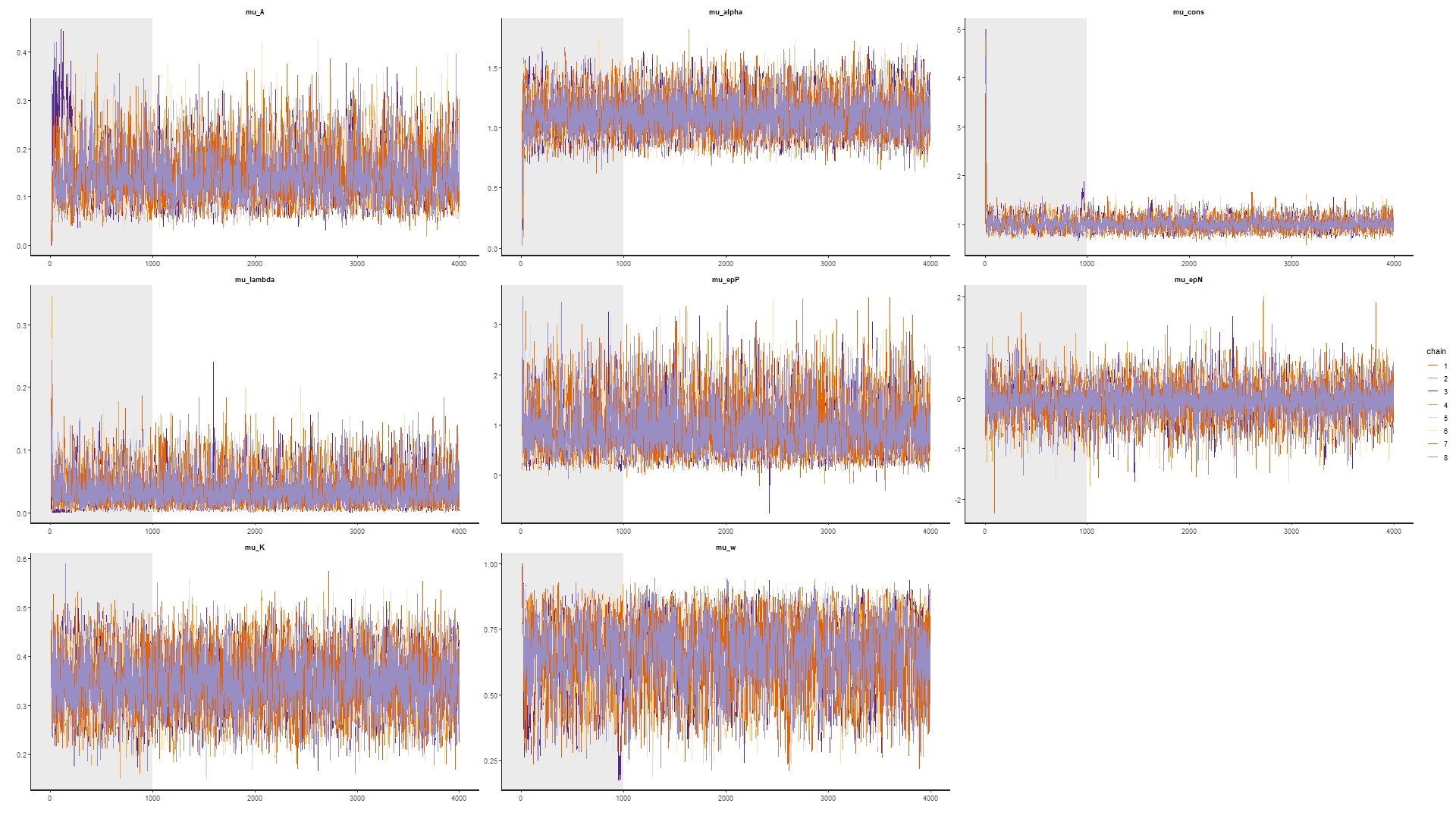


Figure S1 The MCMC performance of hyper parameters with trace plots of methamphetamine use disorder group before rTMS in VPP model, related to Figure 3,4.


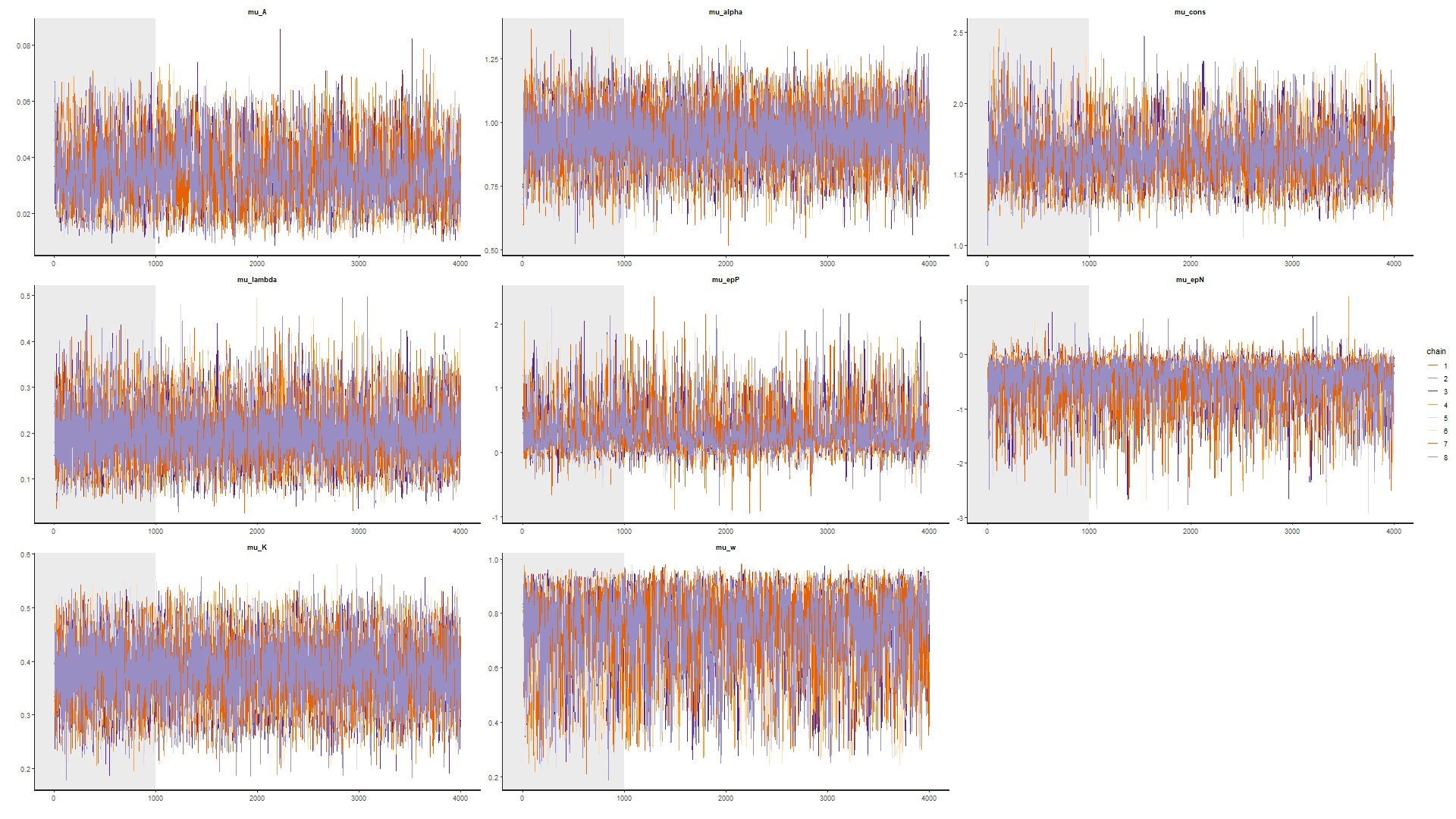


Figure S2 The MCMC performance of hyper parameters with trace plots of health control in VPP model, related to Figure 3.


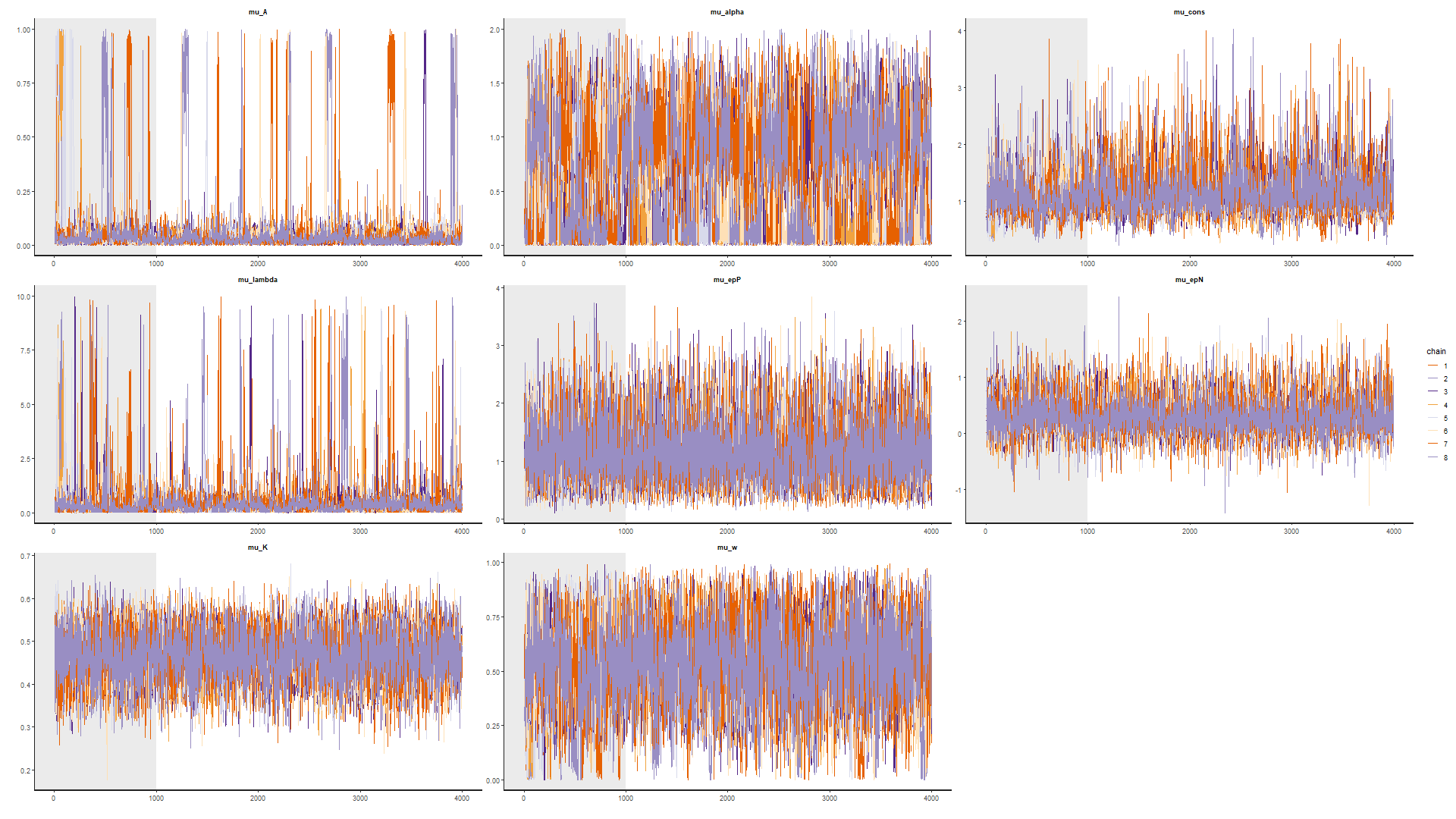


Figure S3 The MCMC performance of hyper parameters with trace plots of methamphetamine use disorder group after rTMS in VPP model, related to Figure 4.


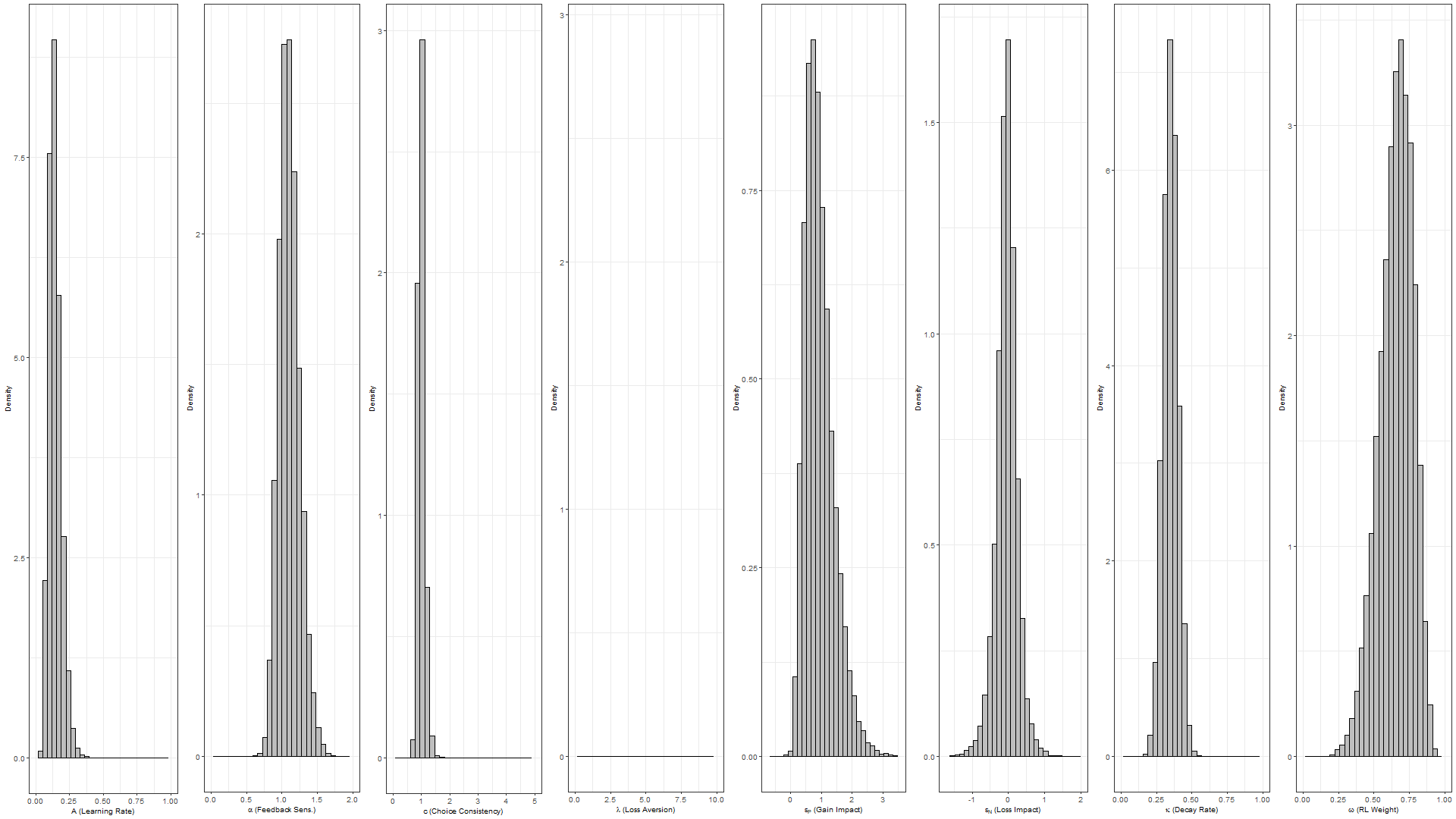


Figure S4 The posterior parameter distribution of methamphetamine use disorder group before rTMS in VPP model, related to Figure 3,4.


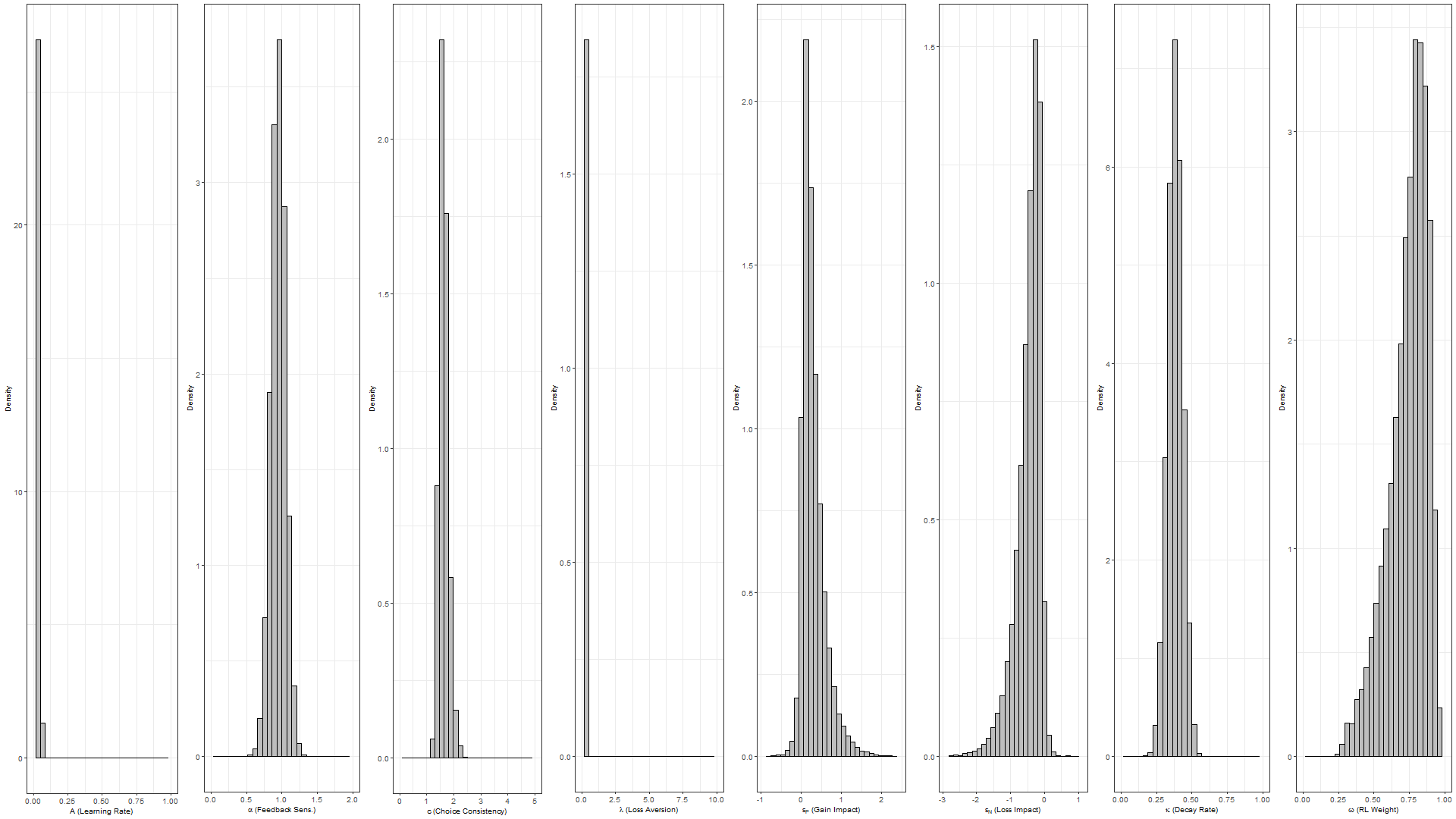


Figure S5 The posterior parameter distribution of health control group in VPP model, related to Figure 3.


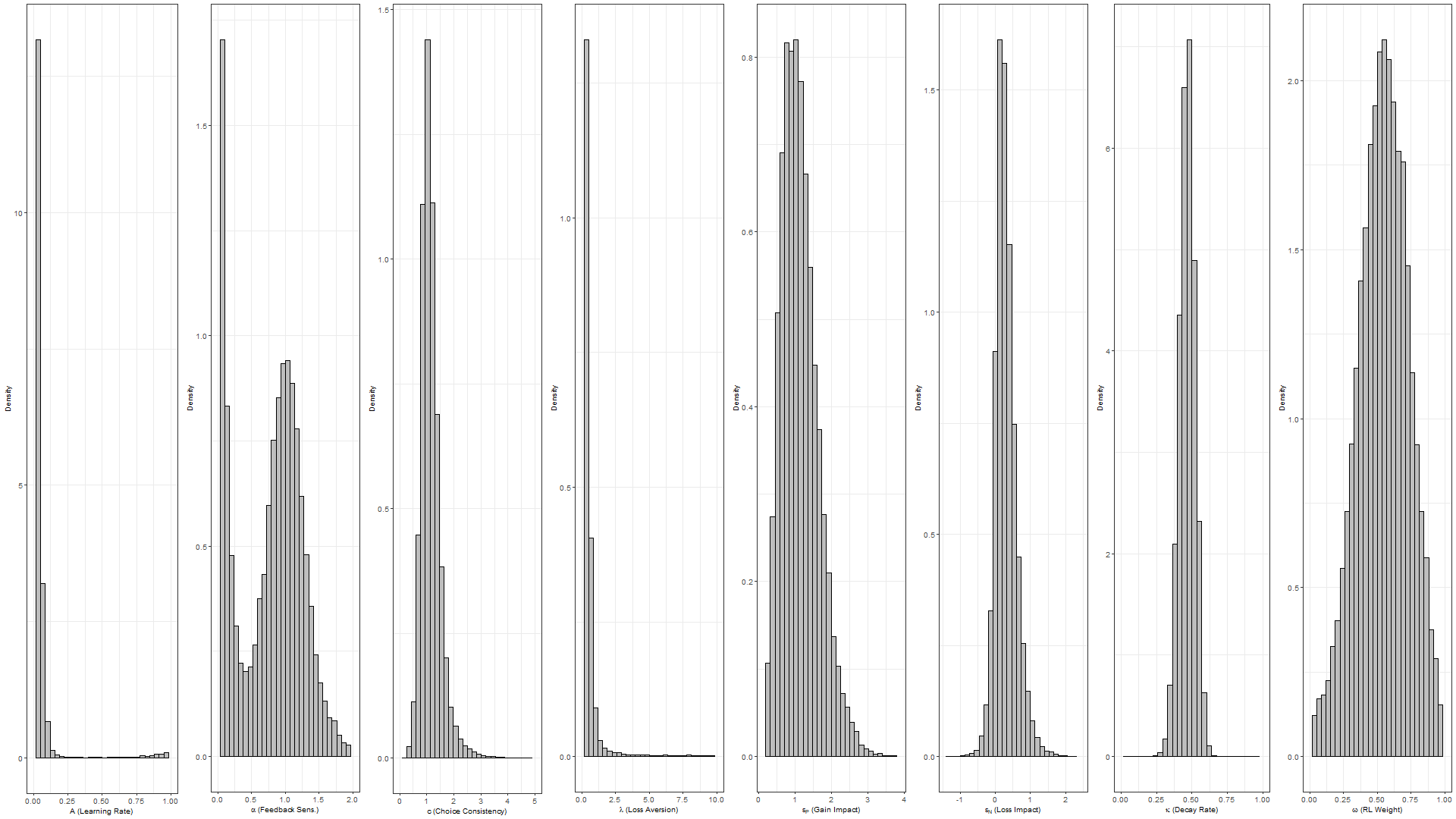


Figure S6 The posterior parameter distribution of methamphetamine use disorder group after rTMS in VPP model, related to Figure 4.


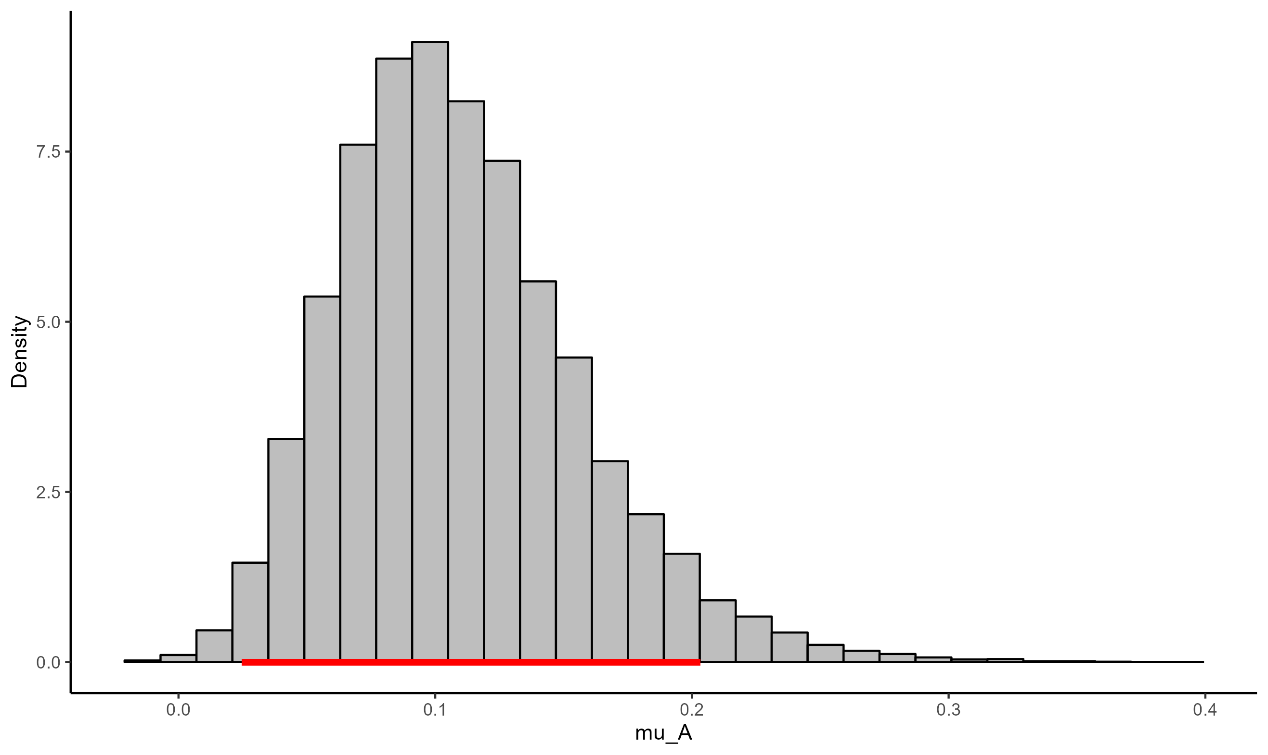


Figure S7 The 95% Highest Density Interval for parameter *A* between methamphetamine use disorder group before rTMS minus health control group, related to Figure 3.


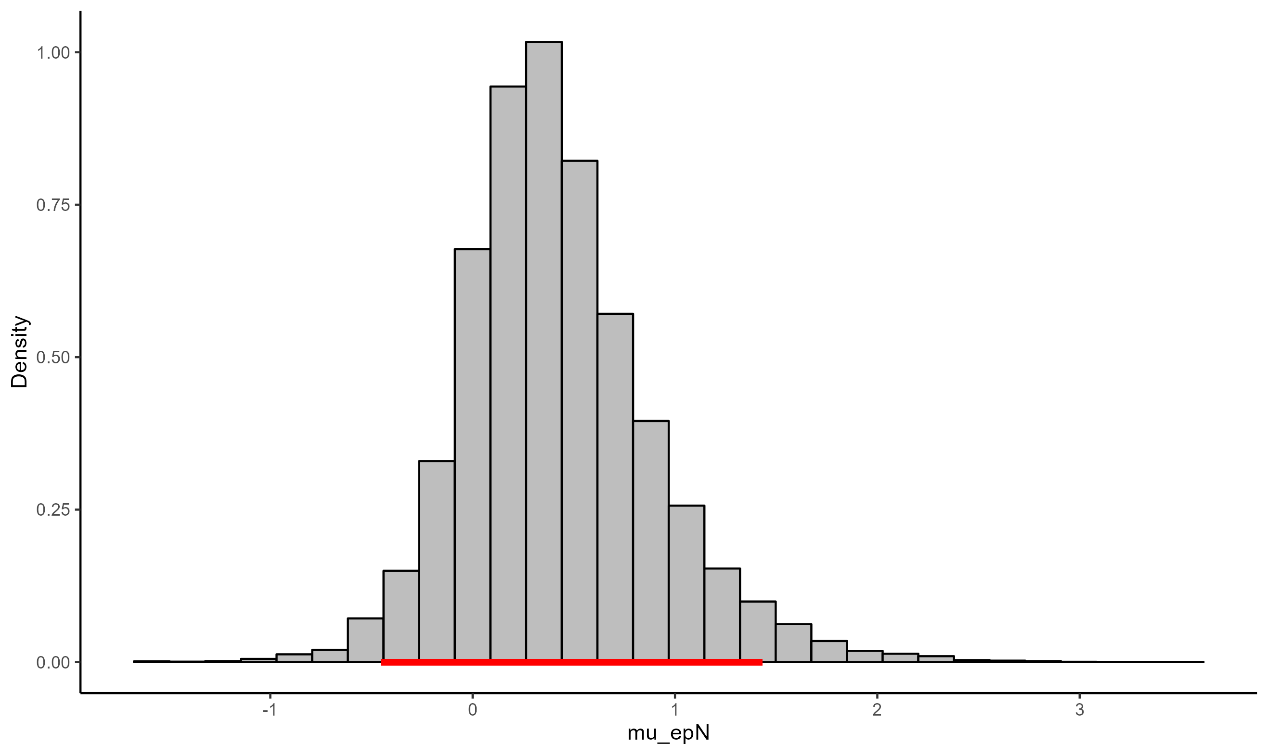


Figure S8 The 95% Highest Density Interval for parameter epN between methamphetamine use disorder group before rTMS minus health control group, related to Figure 3.


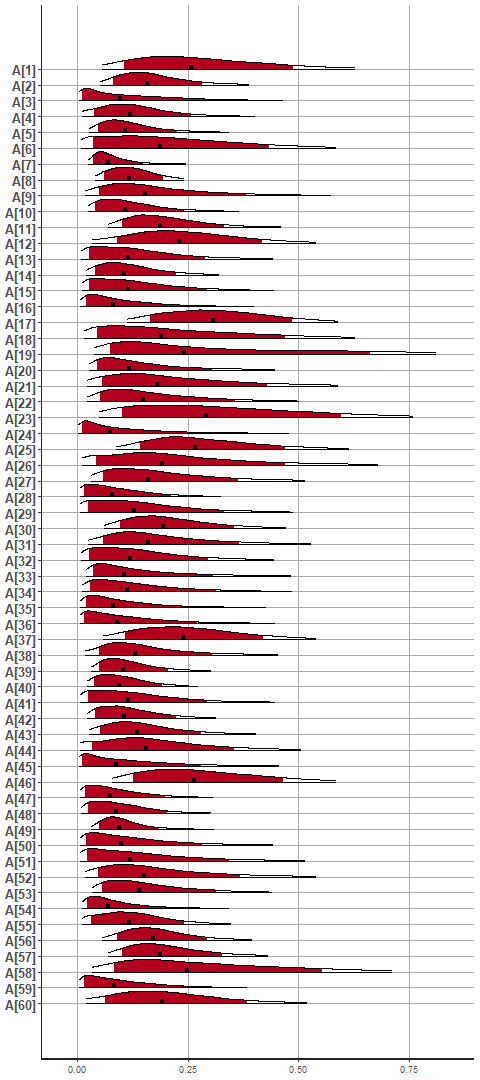


Figure S9 The Density Interval for parameter A in methamphetamine use disorder group before rTMS (ci_level: 0.8 (80% intervals), outer_level: 0.95 (95% intervals)), related to Figure 3.


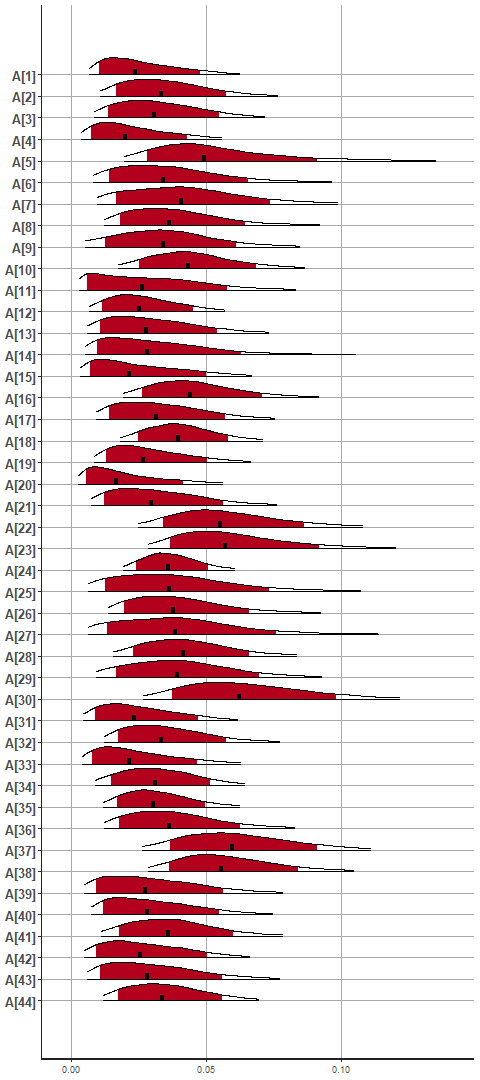


Figure S10 The Density Interval for parameter A in health control group (ci_level: 0.8 (80% intervals), outer_level: 0.95 (95% intervals)), related to Figure 3.

[Bibliography](https://sciwheel.com/work/bibliography)

[Ahn, W.-Y., Vasilev, G., Lee, S.-H., Busemeyer, J. R., Kruschke, J. K., Bechara, A., & Vassileva, J. (2014). Decision-making in stimulant and opiate addicts in protracted abstinence: evidence from computational modeling with pure users. *Frontiers in Psychology*, *5*, 849. https://doi.org/10.3389/fpsyg.2014.00849](https://sciwheel.com/work/bibliography/3930979)

[Vehtari, A., Gelman, A., & Gabry, J. (2017). Practical Bayesian model evaluation using leave-one-out cross-validation and WAIC. *Statistics and Computing*, *27*(5), 1413–1432. https://doi.org/10.1007/s11222-016-9696-4](https://sciwheel.com/work/bibliography/4604910)
